# Development and Validation of Green and High-Throughput Microwell Spectrophotometric Assay for the Determination of Selective Serotonin Reuptake Inhibitors in Their Pharmaceutical Dosage Forms

**DOI:** 10.3390/molecules28104221

**Published:** 2023-05-21

**Authors:** Ibrahim A. Darwish, Nourah Z. Alzoman

**Affiliations:** Department of Pharmaceutical Chemistry, College of Pharmacy, King Saud University, P.O. Box 2457, Riyadh 11451, Saudi Arabia; nalzoman@ksu.edu.sa

**Keywords:** green and high-throughput analysis, microwell assay, pharmaceutical quality control, spectrophotometry, SSRIs

## Abstract

This study describes the development and validation of a new green and high-throughput microwell spectrophotometric assay (MW-SPA) for the determination of three selective serotonin reuptake inhibitors (SSRIs) in their pharmaceutical dosage forms. These SSRIs are fluoxetine, fluvoxamine, and paroxetine, the most prescribed drugs for the treatment of depression. The proposed assay was based on the formation of orange-colored *N*-substituted naphthoquinone derivatives upon the reaction of SSRIs with 1,2-naphthoquinone-4-sulphonate (NQS) in alkaline media. The assay was conducted in 96-microwell assay plates, and the absorbances of the reaction products were measured by a microplate reader at their maximum absorbance wavelengths. The optimum conditions of the reaction were refined and established. Under these conditions, calibration curves were generated, and linear regression equations were computed. The linear relations between the absorbances and drug concentrations were linear with good correlation coefficients (0.9992–0.9997) in the range of 2–80 µg/mL. The assay limits of detection were in the range of 1.5–4.2 µg/mL. The precision was satisfactory as the values of relative standard deviation did not exceed 1.70%. The accuracy of the assay was ≥98.2%. The proposed MW-SPA was successfully applied to the analysis of the SSRIs in their pharmaceutical dosage forms with acceptable accuracy and precision; the label claims were 99.2–100.5% (±0.96–1.35%). The results of the proposed MW-SPA were compared with those of the official/pre-validated assays by statistical analysis with respect to the accuracy (by *t*-test) and precision (by F-test). No significant differences were found between the calculated and theoretical values of the t- and F-tests at the 95% confidence level, proving similar accuracy and precision in the determination of SSRIs by both assays. The greenness of the proposed assay was confirmed by two metric tools. In addition, the assay is characterized with a high-throughput property which enables the simultaneous analysis of many samples in a short time. Therefore, the assay is a valuable tool for rapid routine application in pharmaceutical quality control units for the determination of the investigated SSRIs.

## 1. Introduction

Depression is a prevalent psychiatric or mental disorder that affects approximately 5% of adults worldwide. It is mediated by changes in the levels of some central neurotransmitters and/or their biochemical functions. The change in the balance of these neurotransmitters (norepinephrine, serotonin, or both) occurs due to an impaired synthesis of neurotransmitters, increased breakdown or metabolism, and/or increased pump uptake [1]. The use of antidepressant drugs is the most commonly used approach for the management of depression. These drugs reduce the reuptake of neurotransmitters and also the generation of cyclic adenosine monophosphate, which is involved in the pathogenesis of depression. There are four main classes of antidepressant drugs, namely tricyclic antidepressants, monoamine oxidase inhibitors, selective serotonin reuptake inhibitors (SSRIs), and other miscellaneous antidepressants [2].

SSRIs are mostly prescribed as the first-line treatment of depression and other psychiatric disorders. The wide use of SSRIs is attributed to their safety, potency, and tolerability. SSRIs exert their antidepressant efficacy by selective inhibition of the reuptake of serotonin by the presynaptic receptors. This inhibitory effect leads to an increase in the level of serotonin at the synapse and thus stimulates the central nervous system. The Food and Drug Administration (FDA) has approved nine SSRI drugs for the treatment of depression. These drugs are fluoxetine, fluvoxamine, paroxetine, sertraline, citalopram, escitalopram, vilazodone, dapoxetine, and vortioxetine. These SSRIs have different chemical structures, leading to differences in their physicochemical and pharmacokinetic characteristics [3]. SSRIs have a similar antidepressant potency to that of tricyclic antidepressants; however, SSRIs have better safety and acceptability in patients. In addition, SSRIs have no sedative side effects, are significantly safe in overdose cases, and have a long half-life, which enables their use at once-daily and/or once-weekly dosing. These combined benefits are the supporting factors standing behind the wide use of SSRIs as antidepressant drugs [4].

The remarkably potent and improved safe therapy for depression with SSRIs is principally dependent on the active drug content and content uniformity of their pharmaceutical dosage forms. To ensure these requirements are met, a proper assay is required for quality control of their dosage forms. Different assays relying on varying techniques have been described for the quality control of dosage forms of SSRIs. These assays/techniques were reviewed and published in some review articles [5,6]. Among these assays, spectrophotometric assays are the most widely used, as indicated from the number of published articles describing spectrophotometric assays for SSRIs [7,8,9,10,11,12,13,14,15,16,17,18,19]. The great interest in spectrophotometric assays for SSRIs is due to their simplicity, convenience, low cost, ready automation with spectrophotometric analyzers, and availability of the technique in almost all quality control laboratories in pharmaceutical manufacturing companies [17,18,19]. However, the existing spectrophotometric assays for SSRIs employ the conventional analytical practice, which uses volumetric flasks/cuvettes in the analysis and is conducted in a manual fashion. Accordingly, the throughputs of these assays are limited and do not fulfill the important needs of quality control laboratories for processing batches containing a large number of pharmaceutical formulations [20,21,22]. In addition, these assays consume large volumes of organic solvents, which are costly and have a harmful impact on the environment and, more importantly, on the health of analysts [23,24,25,26]. Therefore, these assays do not meet the needs of pharmaceutical industries for assays with high throughput [20,21,22] and do not apply the principles of the green analytical chemistry (GAC) approach [27].

**Figure 1:**
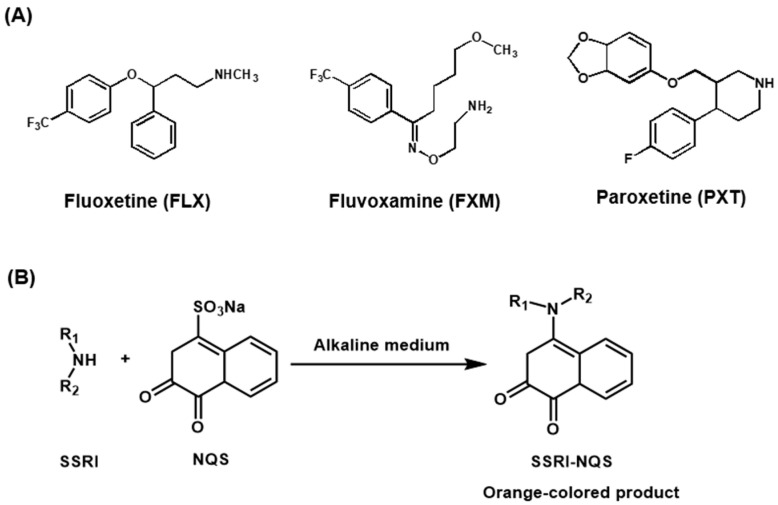
(**A**) The chemical structures of the investigated SSRIs and their abbreviations. (**B**) The chemical reaction of SSRIs with the NQS reagent.

This study describes a new green and high-throughput microwell spectrophotometric assay (MW-SPA) for the quality control of SSRIs in pharmaceutical laboratories. The assay was developed and validated for three SSRIs, which are fluoxetine (FLX), fluvoxamine (FXM), and paroxetine (PXT). These drugs were selected as representative examples of SSRIs because they are the most important and widely prescribed SSRIs in the treatment of depression. These drugs are official in the British Pharmacopoeia [28] and United States Pharmacopeia [29]. The chemical structures of FLX, FXM, and PXT are given in Figure 1, and their chemical names, molecular formulae, and molecular weights are given in Table 1. The assay was based on the reaction of the investigated SSRIs, via their primary or secondary amino groups, with 1,2-naphthoquinone-4-sulphonate (NQS) in an alkaline medium, forming orange-colored *N*-substituted naphthoquinone products. The reaction was conducted in 96-well transparent plates, and the absorbances of the colored reaction products were measured by a microplate reader in absorbance mode. The proposed MW-SPA was proven to meet the principles of the GAC approach and fulfills the demands of high-throughput analysis for the pharmaceutical industry.

## 2. Results and Discussion

### 2.1. Absorption Spectra and Involved Reaction

**Figure 2:**
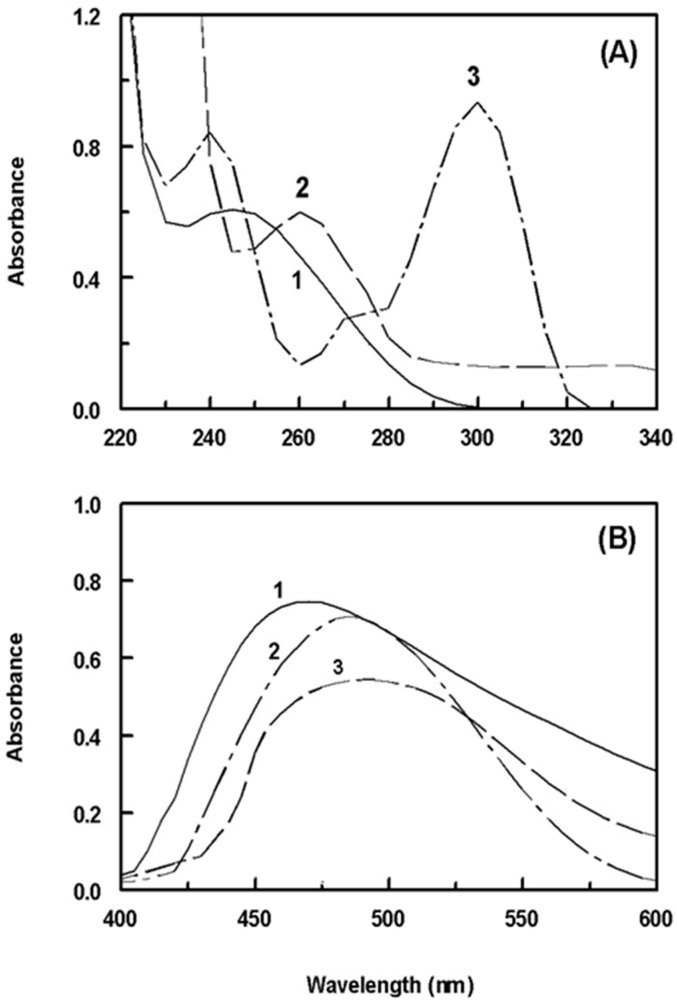
(**A**) The absorption spectra of FXM (1), FLX (2), and PXT (3) against water blanks. The concentrations of FXM, FLX, and PXT were 20, 100, and 150 μg/mL, respectively. (**B**) The absorption spectra of the reaction mixtures of NQS (0.5%, *w*/*v*) with FXM (1), FLX (2), and PXT (3) against reagent blanks. The concentrations of FXM, FLX, and PXT in their reaction mixtures were 20, 15, and 15 μg/mL, respectively.

The absorption spectra of the SSRIs under investigation were recorded against water (Figure 2A). It was observed that the maximum absorption peaks (λ_max_) for FXM, FLX, and PXT were at 245, 260, and 300 nm, respectively. The molar absorptivities (ε) were calculated and found to be 2.1 × 10^3^, 1.3 × 10^3^, and 2.3 × 10^3^ L/mol/cm for FXM, FLX, and PXT, respectively. Due to the significant blue shift of the absorption peaks of the SSRIs, direct measurement of their absorption in the ultraviolet region for determination in dosage forms is susceptible to interferences from co-extracted excipients. Additionally, their low molar absorptivities could result in poor sensitivities. Therefore, derivatization of the SSRIs to more red-shifted light-absorbing derivatives was necessary. For derivatization, the SSRIs were reacted with NQS, forming orange-colored products. The reaction involved condensation of the SSRIs via their primary (FXM) or secondary (FLX and PXT) amino groups with the NQS reagent. The reaction is illustrated in Figure 1B. The absorption spectra of the reaction mixtures were recorded against reagent blanks (Figure 2B). The products had an orange color and exhibited λ_max_ at 470 nm (for FXM) and 490 nm (for FLX and PXT). The λ_max_ of the SSRIs-NQS derivatives was significantly red-shifted by 225, 230, and 190 nm for FXM, FLX, and PXT, respectively, which eliminated the potential interferences. In addition, the ε values were greatly enhanced to 6.1 × 10^4^, 4.8 × 10^4^, and 5.9 × 10^4^ for FXM, FLX, and PXT, respectively, making the assay highly sensitive. Therefore, all subsequent measurements were carried out at 470 nm (for FXM) and 490 nm (for FLX and PXT).

### 2.2. Optimization of Reaction Variables

#### 2.2.1. Effect of NQS Concentration

**Figure 3:**
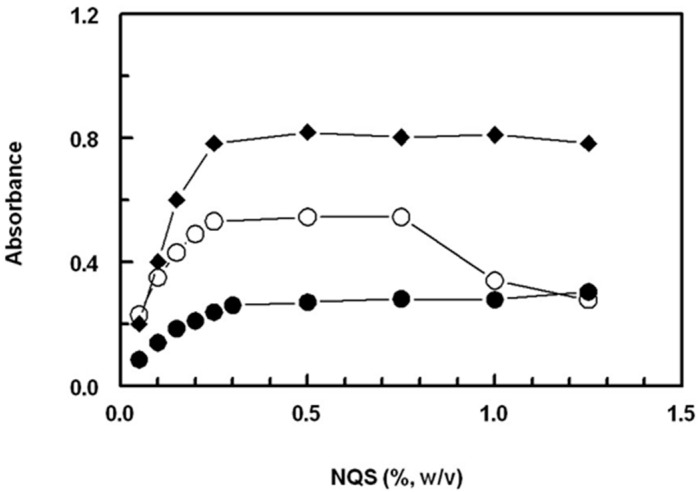
Effect of NQS concentration on its reaction with FXM (●), FLX (○), and PXT (♦). The concentrations of FXM, FLX, and PXT in the reaction mixtures were 10, 10, and 20 μg/mL, respectively.

By studying the impact of the NQS concentration (0.05–1.25%, *w*/*v*) on its reaction with the SSRIs being investigated, it was found that the reactions were dependent on the concentration of NQS. As the NQS concentration increased, the absorbances of the reaction solutions for all SSRIs increased (Figure 3). The highest absorption intensities were observed at an NQS concentration of 0.25% (*w*/*v*). For FXM and PXT, higher concentrations of NQS up to 1.25% (*w*/*v*) had no effect on the absorption values. However, for FLX, NQS concentrations higher than 0.75% (*w*/*v*) led to a decrease in the absorbance values. To obtain more precise readings, further experiments were conducted using NQS at a concentration of 0.5% (*w*/*v*).

#### 2.2.2. Effect of pH

**Figure 4:**
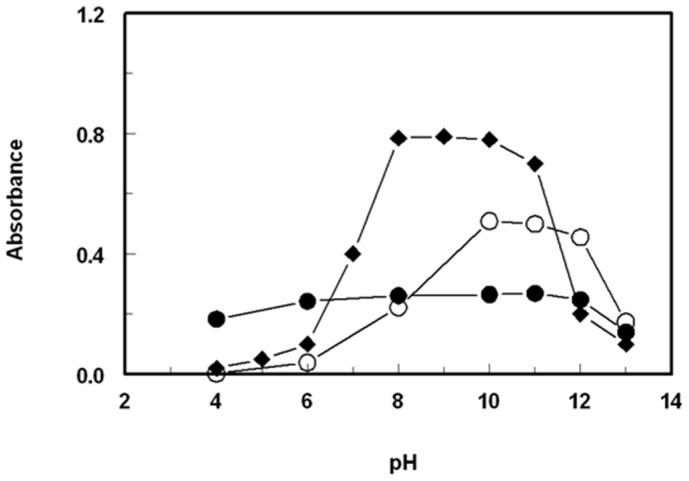
Effect of pH on the reaction of NQS (0.5%, *w*/*v*) with FXM (●), FLX (○), and PXT (♦). The concentrations of FXM, FLX, and PXT in the reaction mixtures were 10, 10, and 20 μg/mL, respectively.

The impact of pH on the absorbance of the SSRIs-NQS products was explored by conducting the reaction in Clark and Lubs buffer solutions with varying pH values (ranging from 4 to 13). The results showed that the absorbances were quite low at acidic pH values, indicating the challenge of the reaction under such conditions (Figure 4). The low reactivity at acidic pH was attributed to the fact that the amino groups of the SSRIs exist in the form of acid salt, which reduces their nucleophilic substitution capabilities. As the pH became alkaline, the absorbances increased rapidly as the amino groups of the SSRIs (in the acid salts) became free, facilitating the nucleophilic substitution reactions. The maximum absorption values were achieved in the pH range of 8–12 for FXM, 10–11 for FLX, and 8–10 for PXT. At higher pH values, the absorbances of the solutions decreased, possibly due to an increase in the concentration of hydroxide ions that hindered the condensation reaction between the SSRIs and NQS. Subsequent experiments were conducted at pH 9 for FXM and PXT and at pH 11 for FLX.

#### 2.2.3. Effect of Temperature

The impact of temperature on the reactions was investigated by allowing the reactions to proceed at different temperatures (ranging from 25 to 50 °C), controlled by the built-in temperature control of the microwell plate reader. It was found that in the case of FLX and PXT, increasing the temperature of the reaction solution did not affect the absorbance values. For FXM, an increase in temperature led to an increase in the absorption values, with the maximum absorbance values attained at 40 °C, but the increase in the absorbance values was not significantly high. To develop a simple assay for all SSRIs, further experiments were conducted at room temperature (25 ± 5 °C) for the three SSRIs.

#### 2.2.4. Effect of Reaction Time

**Figure 5:**
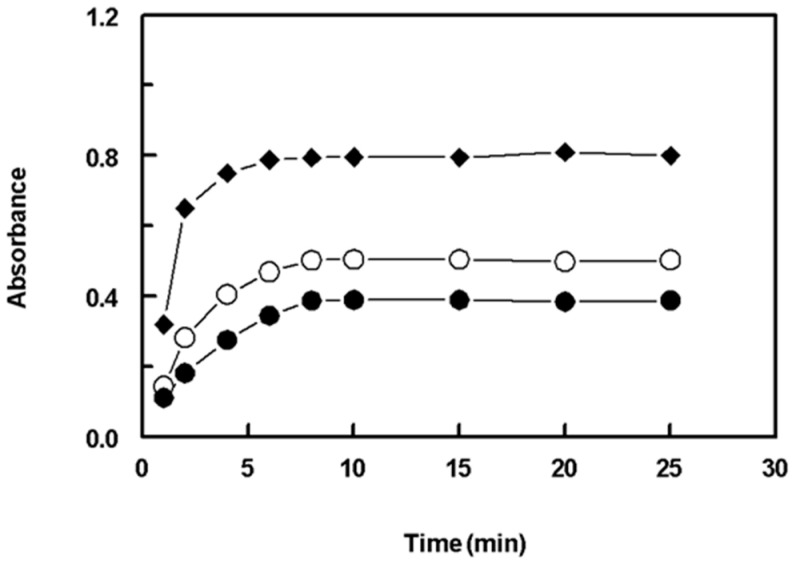
Effect of time on the reaction of NQS (0.5%, *w*/*v*) with FXM (●), FLX (○), and PXT (♦). The concentrations of FXM, FLX, and PXT in the reaction mixtures were 10, 10, and 20 μg/mL, respectively.

The impact of time on the formation of the reaction products was examined by allowing the reactions to proceed for different lengths of time. The findings indicated that the reactions involving the SSRIs were completed in less than 10 min, and longer reaction times of up to 25 min did not have any effect on the reactions (Figure 5). As a result, further experiments were conducted with a reaction time of 10 min.

#### 2.2.5. Effect of Solvent

**Figure 6:**
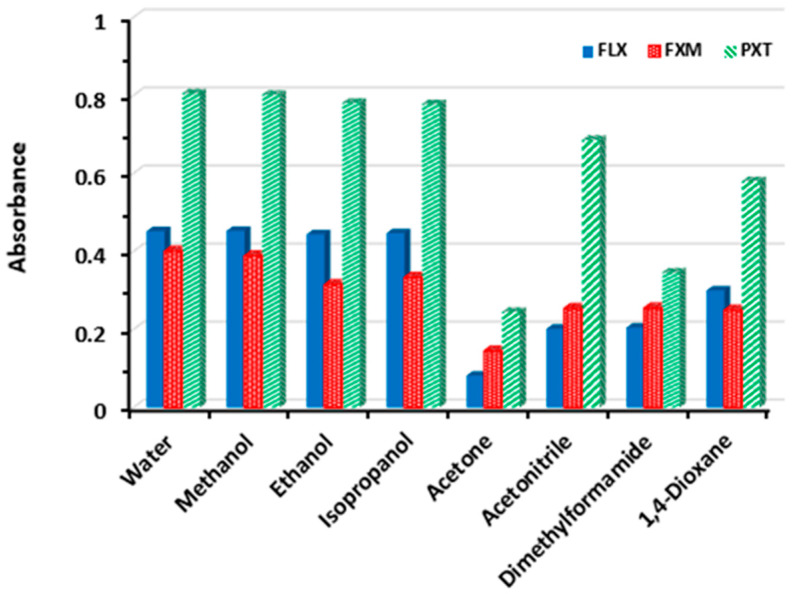
Effect of solvent on the reaction of NQS (0.5%, *w*/*v*) with FXM, FLX, and PXT. The concentrations of FXM, FLX, and PXT in the reaction mixtures were 10, 10, and 20 μg/mL, respectively.

In order to select the most appropriate solvent for carrying out the reaction, the following different solvents were tested: water, methanol, ethanol, isopropanol, acetone, acetonitrile, dimethylformamide, and 1,4-dioxane. The highest absorbance readings were obtained when water, methanol, ethanol, and isopropanol were used, and lower absorbance values were obtained when the other solvents were used (Figure 6). To develop a green and inexpensive assay, water was used as a solvent.

#### 2.2.6. Stability of the Chromogen

Using the optimal conditions identified earlier, the reactions between SSRIs and NQS were completed within 10 min, and the absorbances did not change after 25 min of standing. The stability of the chromogen over time was also examined by measuring the absorption intensity of the reaction solution at various time intervals. The results showed that the absorbance of the chromogen remained stable for at least 2 h, which improved the convenience and applicability of the assay for a large number of samples.

Under the aforementioned optimum conditions, the reactions between SSRIs and NQS were completed within 10 min, and the absorbances no longer changed after standing for up to 25 min. The effect of time on the stability of the chromogen was studied by following the absorption intensity of the reaction solution (after dilution) at different time intervals. It was found that the absorbance of the chromogen remained stable for at least 2 h. This increased the convenience of the method as well as making it applicable for a large number of samples.

A summary of the optimization of the variables affecting the reaction of NQS with the investigated SSRIs is given in Table 2.

### 2.3. Validation of the Proposed MW-SPA

#### 2.3.1. Calibration and Sensitivity

**Figure 7:**
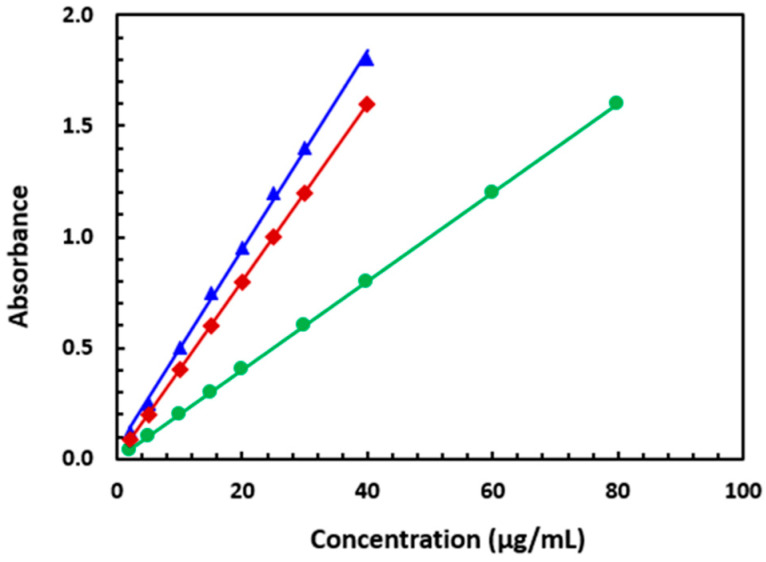
The calibration curves for the determination of FXM (●), FLX (▲), and PXT (♦) by the proposed MW-SPA via their reaction with NQS reagent.

Using the optimal conditions outlined in Table 2, calibration curves for determining SSRIs through their reaction with NQS were constructed by plotting absorbances against their concentrations (Figure 7). The regression equation was A = a + bC, where A represents the absorbance at λ_max_ and C represents the concentration of the SSRI in µg/mL. Linear relations with small intercepts (0.0015–0.0502) and high correlation coefficients (0.9992–0.9997) were obtained for the three SSRIs in the concentration range of 5–80 µg/mL for FXM and 2–40 µg/mL for FLX and PXT. The limits of detection (LODs) and limits of quantification (LOQs) were determined according to the International Conference of Harmonization (ICH) guidelines for validating analytical procedures [30]. The following formula was used: LOD or LOQ = κSDa/b, where κ = 3.3 for LOD and 10 for LOQ, SDa is the standard deviation of the intercept, and b is the slope. The LOD values were 4.2, 1.5, and 1.8 µg/mL for FXM, FLX, and PXT, respectively. The LOQ values were 12.7, 4.5, and 5.5 µg/mL for FXM, FLX, and PXT, respectively.

The parameters for the analytical performance of the proposed method are summarized in Table 3.

#### 2.3.2. Reproducibility and Accuracy

To assess the reproducibility of the proposed MW-SPA, five separate solutions of the working standard SSRI solutions were analyzed. The assay showed satisfactory reproducibility, as the relative standard deviation (RSD) did not exceed 1.70% (Table 4). This precision level is sufficient for the routine analysis of the investigated drugs in quality control laboratories.

To assess the accuracy of the proposed assay, recovery studies were conducted by adding known concentrations of the SSRIs to the sample and analyzing them using the proposed assay. The recovery values were found to be within the range of 97.8–102.2 ± 0.56–1.94% (Table 5), indicating the accuracy of the assay.

#### 2.3.3. Robustness and Ruggedness

To assess the robustness of the assay, the impact of small variations in the assay variables on its analytical performance was evaluated. In these experiments, one parameter was changed while keeping the others constant, and the recovery percentage was calculated each time. The results showed that slight variations in the method variables did not have a significant impact on the procedure, as the recovery values ranged between 97.5–101.5 ± 0.78–1.87% (Table 6). This indicates the reliability of the proposed assay during routine analysis of SSRIs.

Ruggedness was also tested by applying the proposed assay to the analysis of SSRIs under the same operational conditions by two independent analysts on three different days. The results obtained from analyst-to-analyst and day-to-day variations were reproducible, with the RSD not exceeding 1.74% (Table 7).

### 2.4. Application of the Proposed MW-SPA

The results mentioned above demonstrate that the proposed assay produced satisfactory results when used to analyze bulk SSRIs. Therefore, the SSRI contents in their pharmaceutical dosage forms were analyzed using both the proposed assay and the official methods [28,29]. The label claim percentages obtained using the proposed method were 99.2–101.3 ± 0.96–1.61% (Table 8). To compare the results with those obtained from the official assay, statistical analysis was performed to assess accuracy (using a *t*-test) and precision (using an F-test). The results showed no significant differences between the calculated and theoretical values of the *t*- and F-tests at a 95% confidence level, indicating similar accuracy and precision in the determination of SSRIs by both assays.

### 2.5. Greenness of the Proposed MW-SPA

Generally, assays involving microwell plates and microplate readers mostly follow the principles of GAC. This is attributed to the fact that these assays consume small volumes of solvents/reagents and produce low volumes of waste. To accurately assess the greenness of the proposed MW-SPA for SSRIs, two different metric tools were used. These tools were GAPI [31] and AGREE [32].

The GAPI metric tool [31] is capable of providing a reliable and comprehensive assessment of the ecological impact of an entire analytical procedure based on 15 parameters classified into five categories. These categories cover all aspects of the analytical process, including sample handling, sample preparation, the use of solvents/reagents, instrumentation, and the general type of assay. The results are displayed as a pictogram consisting of 15 sections, each assigned a color (green, yellow, or red). The green color indicates a safe procedure, while the red color indicates a non-green procedure.

The AGREE metric tool [32] is a new method for assessing the greenness of analytical procedures. This tool employs user-friendly software that yields comprehensive, flexible, informative, and easily interpretable results. The AGREE software evaluates 12 parameters based on the principles of GAC. The results are automatically generated and presented as a circular pictogram consisting of 12 sections, each assigned a specific color ranging from deep green (score = 1) to deep red (score = 0). The overall score, which is a fraction of unity, is automatically calculated and displayed in the center of the pictogram.

**Figure 8:**
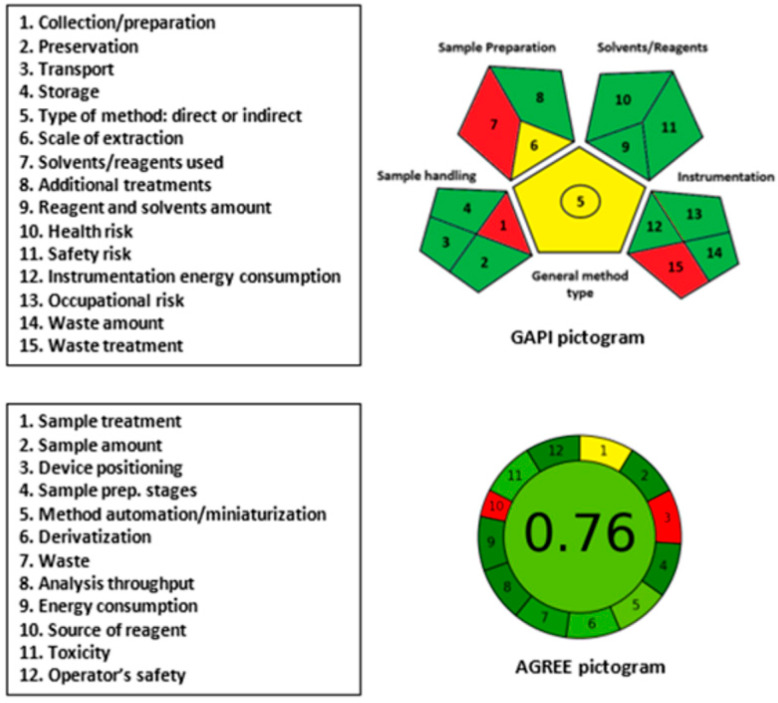
Results of GAPI and AGREE analyses for evaluation of the greenness of the proposed MW-SPA for SSRIs.

In the GAPI pictogram (Figure 8), three parameters (1, 7, and 15) related to sample collection/preparation (1), the use of solvents/reagents (7), and waste treatment (15) are shown in red. This is due to the use of an off-line sample collection/preparation, the use of buffer and NQS reagent, and the lack of intrinsic treatment for the assay’s waste, respectively. It is worth noting that the university has an integrated system for the safe disposal of all organic/hazardous substances. However, the waste treatment parameter of the proposed assay was shown in red to provide a fair assessment of the assay, regardless of the integrated university system.

In the AGREE pictogram (Figure 8), parameters number 3 (device positioning) and 10 (source of reagents) took red colors because the sample treatment was carried out off-line and the microplate reader was not operated in an automated way. The other parameters took yellow or varying degrees of green colors. The automatically generated number in the center of the AGREE pictogram for overall assessment was 0.76 out of 1, which confirmed the overall greenness of the proposed assay.

In conclusion, the overall evaluation of greenness of the proposed MW-SPA fulfills the requirements for GAC for routine application in pharmaceutical quality control laboratories for analysis of the investigated SSRIs.

## 3. Experimental

### 3.1. Instruments

The SpectraMax^®^ M5 microplate reader was manufactured by Molecular Devices, LLC (San Jose, CA, USA). The reader is controlled by SoftMax^®^ Pro Enterprise software (version 7.1), which was provided with the reader. Additionally, the JB1603-C/FACT digital balance is a product of Mettler-Toledo International Inc., located in Zürich, Switzerland. All spectrophotometric spectra were recorded using a V-530 ultraviolet-visible spectrophotometer manufactured by JASCO Co., Ltd. (Kyoto, Japan). The pH meter used in the experiments was the Model Jenway 350 m, manufactured by Bibby Scientific Ltd. (Essex, UK).

### 3.2. Standard Materials and Dosage Forms

The standards used in the study, namely fluoxetine HCl (FLX), fluvoxamine maleate (FXM), and paroxetine HCl (PXT), were obtained from their respective manufacturers, namely Hetero Drugs Ltd. in Hyderabad, India, for FLX; Solvay Pharma in Suresnes, France, for FXM; and SmithKline Beecham Pharmaceuticals in Brentford, England, for PXT. The appearance and solubility of the drugs were described before [28,29]. The dosage forms of FLX, FXM, and PXT used in this investigation with their manufacturers and strengths (mg/unit; tablet or capsule) are summarized in Table 9.

### 3.3. Reagents, Buffer Solutions, and Tools

1,2-Naphthoquinone-4-sulphonate (NQS) was purchased from Sigma-Aldrich Chemicals Co. (St. Louis, MI, USA). The NQS solution was 0.5% (*w*/*v*, in water). Clark and Lubs buffer solutions of different pH values were used. Transparent microwell plates with 96 wells were acquired from Corning/Costar Inc. (Cambridge, MA, USA). Variable volumes and adjustable multi-channel pipettes were purchased from Sigma-Aldrich Chemicals Co. (St. Louis, MI, USA). The solvents used in the study were spectroscopic-grade and were obtained from Fisher Scientific (California, CA, USA).

### 3.4. Preparation of Standard and Sample Solutions

#### 3.4.1. Standard Solutions

An accurate weight (20 mg) of each drug was dissolved in 10 mL distilled water to obtain a stock solution of 2 mg/mL. An aliquot (1 mL) of the stock solution was diluted with water to obtain working solutions in the range of 2–40 µg/mL for FLX and PXT and in the range of 5–80 µg/mL for FXM.

#### 3.4.2. Dosage form Sample Solutions

To prepare the sample, either 10 tablets or the contents of 10 capsules were weighed and ground into a fine powder. An accurately weighed amount of the powder, equivalent to 50 mg of the active ingredient, was dissolved in 50 mL of distilled water. The resulting solution was filtered. An aliquot of the filtrate (1 mL) was then diluted with distilled water to obtain appropriate concentrations for analysis within the linear range of each SSRI.

### 3.5. General Procedure of MW-SPA

Aliquots (100 µL) of SSRI solution containing concentrations in the range of 2–40 µg/mL for FLX and PXT and 5–80 µg/mL for FXM were transferred into separate wells of the 96-well assay plates. Then, 50 µL of buffer solution of pH 9 for FXM and PXT and of pH 11 for FLX was added, followed by 50 µL of NQS solution (0.5%, *w*/*v*). The reaction solution was allowed to proceed for 10 min at room temperature (25 ± 5 °C). The absorbance of the solution in each well was measured by the microplate reader at 470 nm for FXM and at 490 nm for FLX and PXT. The blank wells were treated in a similar way, except 100 µL of chloroform was dispensed in each well instead of the standard drug solution.

## 4. Conclusions

The current study presents the development and validation of an MW-SPA for three SSRIs, namely FLX, FXM, and PXT. The proposed assay employs the formation of colored products upon the condensation reactions of the SSRIs, via their amino groups, with NQS reagent. The proposed assay offers several advantages over previously reported spectrophotometric assays for SSRIs. These advantages include the use of low volumes of samples/solvents (which is cost-effective and environmentally friendly), ease of use, and high throughput (making it suitable for the pharmaceutical industry). The assay is useful for the routine analysis of SSRIs in dosage forms with satisfactory accuracy and precision, and it also expands the utilization of microwell assays assisted with microplate readers for quantifying various pharmaceuticals in the pharmaceutical industry. The limitation of the proposed assay is its application to the analysis of the cited SSRIs in their biological fluids. Current work is going to develop microwell assays for the determination of the cited SSRIs in biological samples.

## Figures and Tables

**Table 1:** The names, chemical nomenclature, molecular formulae, and molecular weights of the investigated SSRIs.

Drug Name (Abbreviation)	Chemical Nomenclature	Molecular Formula	Molecular Weight
Fluoxetine (FLX)	(*3RS*)-*N*-methyl-3-phenyl-3-[4-(trifluoromethyl)phenoxy] propan-1-amine, as hydrochloride	C_17_H_18_F_3_NO.HCl	345.8
Fluvoxamine (FXM)	(*E*)-5-methoxy-4′-trifluromethylvalerophenone O-2-aminoethyloxime, as maleate	C_15_H_21_F_3_N_2_O_2_. C_4_H_4_O_4_	434.4
Paroxetine (PXT)	(3*S*,4*R*)-3-(1,3-benzodioxol-5-yloxymethyl)-4-(4-fluorophenyl)piperidine, as hydrochloride	C_19_H_20_FNO_3_.HCl	365.8

**Table 2:** Summary of the optimization of variables affecting the reaction of NQS with the investigated SSRIs.

Variable	Studied Range	Optimum Condition		
		FXM	FLX	PXT
Measuring wavelength (nm)	400–600	470	490	490
NQS (%, *w*/*v*)	0.05–1.25	0.5	0.5	0.5
pH	4–13	9	11	9
Temperature (°C)	25–50	40 ^a^	25	25
Time (min)	2–25	10	10	10
Solvent	Different ^b^	Water	Water	Water
Stability of chromogen (hour)	0.5–2	2	2	2

^a^ The subsequent experiments involved in assay procedures were carried out at 25 °C. ^b^ Solvents tested: water, methanol, ethanol, isopropanol, acetone, acetonitrile, dimethyl sulfoxide, and 1,4-dioxane.

**Table 3:** Statistical parameters for the determination of SSRIs by the proposed MW-SPA based on their reaction with NQS.

Parameter	FXM	FLX	PXT
λ_max_ (nm)	470	490	490
Linear range (μg/mL)	5–80	2–40	2–40
Intercept	0.0061	0.0502	0.0015
SD of intercept	0.0253	0.0201	0.0225
Slope	0.0199	0.0442	0.0412
SD of slope	0.0054	0.0091	0.0211
Correlation coefficient (r)	0.9996	0.9997	0.9992
LOD (μg/mL)	4.2	1.5	1.8
LOQ (μg/mL)	12.7	4.5	5.5

**Table 4:** Replicate analysis of SSRIs by the proposed MW-SPA for the determination of SSRIs by the proposed MW-SPA.

Sample Number	Absorbance		
	FXM (20 μg/mL)	FLX (20 μg/mL)	PXT (20 μg/mL)
1	0.453	0.953	0.804
2	0.458	0.958	0.815
3	0.451	0.951	0.799
4	0.471	0.971	0.815
5	0.459	0.959	0.799
Mean	0.458	0.958	0.806
SD	0.008	0.008	0.008
RSD (%)	1.70	0.81	1.01

**Table 5:** Recovery studies for determination of SSRIs by the proposed MW-SPA.

SSRI Concentration (μg/mL)	Recovery (% ± SD) ^a^		
	FXM	FLX	PXT
5	101.0 ± 1.94	98.8 ± 0.56	99.5 ± 1.85
10	99.3 ± 1.54	99.5 ± 1.04	98.9 ± 1.20
15	98.5 ± 0.87	101.5 ± 1.15	101.3 ± 0.92
20	101.6 ± 1.54	102.2 ± 0.95	97.8 ± 0.84
25	98.2 ± 1.86	99.0 ± 0.76	99.7 ± 1.50

^a^ Values are the mean of three determinations.

**Table 6:** Robustness of the proposed MW-SPA for determination of SSRIs by their reaction with NQS reagent.

Parameters	Recovery (% ± SD) ^a^		
	FXM	FLX	PXT
Recommended conditions ^b^	99.2 ± 1.45	98.5 ± 1.45	101.2 ± 1.23
NQS concentration (%, *w*/*v*)			
0.25	100.1 ± 1.36	99.5 ± 1.74	98.6 ± 0.93
0.75	99.4 ± 1.57	97.9 ± 1.45	101.4 ± 1.05
Buffer solution (pH)			
8.8	98.2 ± 1.26	ND ^c^	98.4 ± 1.65
9.2	99.4 ± 0.89	ND	101.2 ± 1.49
10.8	ND	98.9 ± 1.87	ND
11.2	ND	101.5 ± 1.67	ND
Reaction time (min)			
5	97.5 ± 1.58	99.4 ± 1.62	98.9 ± 1.57
15	99.8 ± 1.60	101.1 ± 1.42	100.5 ± 0.78

^a^ Values are the mean of three determinations. ^b^ The recommended conditions are given in the Experimental section. ^c^ ND: not determined.

**Table 7:** Ruggedness of the proposed MW-SPA for determination of SSRIs.

Parameters	Recovery (% ± RSD) ^a^		
	FXM	FLX	PXT
Analyst-to-analyst			
Analyst-1	99.4 ± 0.91	99.7 ± 1.62	99.4 ± 0.96
Analyst-2	96.6 ± 0.82	101.1 ± 1.74	98.5 ± 1.24
Day-to-day			
Day-1	100.4 ± 1.33	100.2 ± 0.62	101.1 ± 1.36
Day-2	98.7 ± 0.92	99.7 ± 1.23	100.5 ± 1.54
Day-3	101.6 ± 1.45	99.8 ± 1.07	99.4 ± 1.25

^a^ Values are the mean of three determinations ± SD.

**Table 8:** Determination of SSRIs in their pharmaceutical dosage forms using the proposed MW-SPA and the official assays.

Product	Label Claim (%) ± SD ^a^		F-Value ^b^	t-Value ^b^
	Proposed Assay	Official Assay ^c^		
Prozac capsules	99.8 ± 1.28	99.6 ± 1.22	1.00	1.10
Fluzac capsules	100.1 ± 1.61	99.7 ± 1.43	1.64	1.27
Salipax capsules	99.8 ± 1.24	100.2 ± 0.85	2.35	2.13
Flutin capsules	100.5 ± 0.96	100.4 ± 0.94	1.02	3.47
Octozac capsules	99.2 ± 1.06	101.3 ± 0.48	2.20	4.88
Faverin tablets	99.9 ± 1.35	100.3 ± 1.25	1.68	1.17
Seroxate tablets	100.1 ± 1.26	99.6 ± 1.12	2.62	1.27

^a^ Values are the mean of five determinations ± SD. ^b^ Theoretical values for t- and F-tests at 95% confidence limit (*n* = 5) were 2.78 and 6.39, respectively. ^c^ Reference [28] for FXM and reference [29] for FLX and PXT.

**Table 9:** The pharmaceutical dosage forms of the investigated SSRIs.

Brand Name (Dosage Form)	Manufacturer (Address)	Active Ingredient (SSRI Drug)	Label Claim (mg/unit)
Prozac capsules	Eli Lilly & Co., Ltd. (Hampshire, UK)	FLX	20
Fluzac capsules	Riyadh Pharma (Riyadh, Saudi Arabia)	FLX	20
Salipax capsules	Mepha Ltd. (Aesch-Basilea, Switzerland)	FLX	20
Flutin capsules	Egyptian International Pharmaceutical Industries Co. (Cairo, Egypt)	FLX	20
Octozac capsules	October Pharma, S.A.E. (Cairo, Egypt)	FLX	20
Faverin tablets	Solvay Pharma (Suresnes, France)	FXM	50
Seroxate tablets	GSK plc, SmithKline Beecham Pharmaceuticals (London, UK)	PAR	20

## Data Availability

All data are available in the article.
